# phylotree.js - a JavaScript library for application development and interactive data visualization in phylogenetics

**DOI:** 10.1186/s12859-018-2283-2

**Published:** 2018-07-25

**Authors:** Stephen D. Shank, Steven Weaver, Sergei L. Kosakovsky Pond

**Affiliations:** Department of Biology and Institute for Genomics and Evolutionary Medicine, Temple University, Philadelphia, 19122 USA

**Keywords:** Phylogenetics, JavaScript, Data visualization, D3

## Abstract

**Background:**

While several JavaScript packages for visualizing phylogenetic trees exist, most are best characterized as frameworks that are designed with a specific set of tasks in mind. Extending such packages to use cases that are not available as features often ends up being difficult. Moreover, existing packages tend to produce standalone widgets that are not designed to serve as middleware, as opposed to flexible tools that can integrate with other components of an application.

**Results:**

phylotree.js is a library that extends the popular data visualization framework d3.js, and is suitable for building JavaScript applications where users can view and interact with phylogenetic trees. The effects of such interactions can be captured and communicated to other package components, making it possible to engineer complex and responsive applications that include phylogenetic trees. phylotree.js implements several abstractions in addition to features, and comes with a documented application programming interface, thus promoting interoperability and extensibility. Example applications include a tool to visualize and annotate phylogenetic trees, a web application for comparative sequence analysis, a structural viewer that interacts with a large phylogenetic tree, and an interactive tanglegram.

**Conclusions:**

phylotree.js is a useful tool and application module for a variety of computational biology software applications. The code is available on Github and is released under the MIT license.

**Electronic supplementary material:**

The online version of this article (10.1186/s12859-018-2283-2) contains supplementary material, which is available to authorized users.

## Background

Ever since Darwin provided one of the first illustrations of a phylogenetic tree in *On the Origin of Species* [1], biologists have used them to convey relationships between organisms, genes, and other biological entities. Several phylogenetic tree packages written in the JavaScript programming language have been developed over the past few years [2–5], providing useful resources for viewing phylogenies on the web. However, most of these packages appear to have been developed with a specific use case in mind. For instance, Phylo.io [4] was created for the purpose of viewing and comparing large phylogenetic trees. On the other hand PhyD3 [3] implements a wide variety of popular phylogenetic visualizations, but has not demonstrated the ability to interface with other components of an application.

Though existing tools excel at achieving their intended purpose, a library that allows users to visualize and interact with trees as a component of a larger application has not been published to our knowledge. In addition to providing a robust set of features out of the box, phylotree.js aims to fill this gap by implementing the appropriate abstractions that allow building user interfaces involving branch selection and responding to these selections in other areas of an application. phylotree.js also provides a well-documented application programming interface (API) and a gallery of examples to help developers write phylogenetic applications and novel interactive data visualizations. A live demo for viewing and annotating trees is available [6].

## Implementation

phylotree.js is written as a d3.js [7] layout. d3.js is a popular JavaScript library for interactive data visualization within the browser and on the web. phylotree.js aims to adhere to d3.js’s philosophy of allowing users to bind data to graphical elements and manipulate them based on user interactions, allowing for a more engaging and informative experience. phylotree.js ships with a collection of examples that demonstrate how to provide a rich experience with as little as five lines of code, and how predefined options can be utilized to toggle common features. However, phylotree.js’s strength lies in its ability to interface with other components, such as those created by d3.js, or other JavaScript packages with appropriate interfaces that they seek to connect with. Furthermore, instead of attempting to service a large but fixed variety of possible use cases, phylotree.js’s design permits customization by providing several abstractions most commonly associated with phylogenetic tree construction and manipulation.

A key abstraction for interoperability with external software and packages is the selection_callback method. This method accepts a function that is called on the current branch selection whenever this selection is updated by the user. Mechanisms for making selections include the ability to select clades, paths to the root node, individual branches, external or internal branches, and branches that are nearby on the screen. Multiple types of selection categories are supported to facilitate comparative analysis. phylotree.js also supports an algorithmic abstraction via the traverse_and_compute method, which allows developers to traverse the tree in either pre- or post-order and compute associated metadata as they proceed. Abstractions such as these allow developers to adhere to JavaScript functional programming, defining custom functions to achieve effects that are not specified in advance, thus greatly promoting extensibility outside of a fixed range of use cases.

phylotree.js also comes with a variety of features that make up common use cases. Cladogram and radial layouts are available. Trees can be ladderized to reveal phylogenetic information that would otherwise be obscured. Edge and node displays are customizable. Nodes are clickable with a pop-up menu that can be customized and extended. Subtrees and clades can either be hidden entirely, or “collapsed” to a spline interpolation of their boundaries so that not all hierarchical information regarding the topology is lost. The level of interactivity is also configurable. Support for Newick format and certain ad hoc extensions, such as those used by HyPhy [8] and BEAST [9], as well as PhyloXML [10] and NeXML [11] are included. In this regard, phylotree.js has many of the features of existing packages already built-in.

By having a variety of built-in features and several core abstractions, phylotree.js has a demonstrable ability to allow to users to select portions of a tree in a wide variety of ways and interface these selections with downstream components of an application. Existing tools can perhaps be characterized as implementing a phylogenetic grammar of graphics, allowing users to create a wide but ultimately fixed variety of visualizations. While this suffices for certain projects (for instance, those that display the results of a large-scale data analysis), most packages have not demonstrated the ability to serve as middleware. phylotree.js is capable of serving as a “glue” component between parts of an application, as demonstrated in the following section.

## Results and discussion

We give three examples of how phylotree.js can be used. First, we describe its use in Datamonkey [12], a webserver for comparative analysis of sequence alignments. Its primary aim is to serve as a user-friendly frontend to HyPhy, a software package for molecular evolution and phylogenetics [8]. HyPhy is capable of fitting phylogenetic models to genetic sequence data; one such example is RELAX [13], which is designed to detect changes in selective pressure across a phylogeny. Branches are split into test and reference sets, and a formal statistical test is carried out to test for selection relaxation on the test set relative to the reference set.

**Fig. 1:**
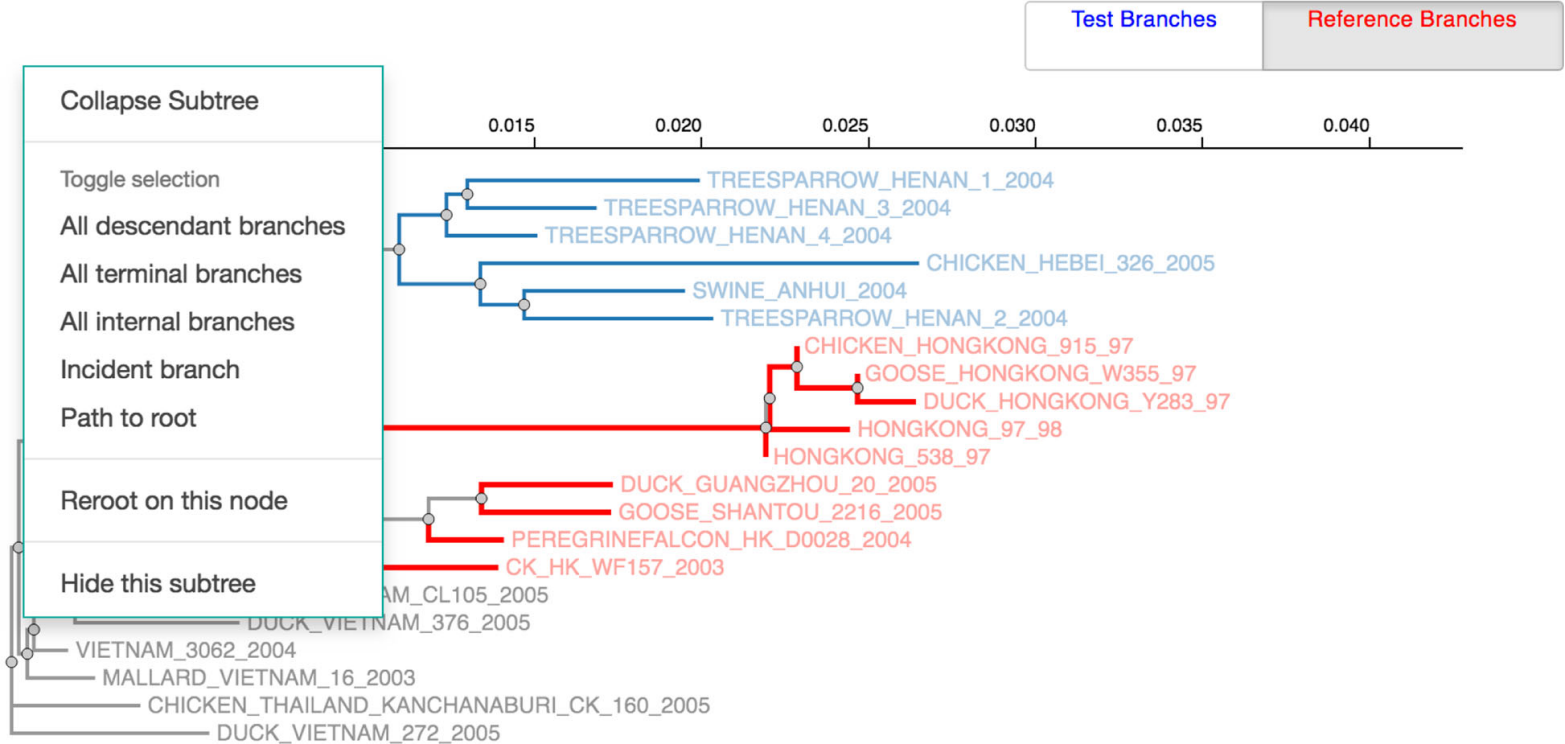
Phylotree as used in Datamonkey. First, a user selects an analysis and uploads a tree. Next, she selects branches for evolutionary hypothesis testing. The tree, having been annotated, is then sent to a high-performance computing cluster along with other data for analysis

Datamonkey provides a graphical user interface to select branches for hypothesis testing in RELAX, depicted in Fig. 1. The user may upload a tree, which is then visualized in the browser using phylotree.js. A user interface is created to enable manual selection of test or reference branches. Once their selection is complete, the tree is serialized to a string in an extended Newick format that encodes the selected annotations and is recognizable by HyPhy on the backend. This example was chosen to illustrate that phylotree.js is capable of serving as a user interface component whose output is consumed downstream in a larger application.

**Fig. 2:**
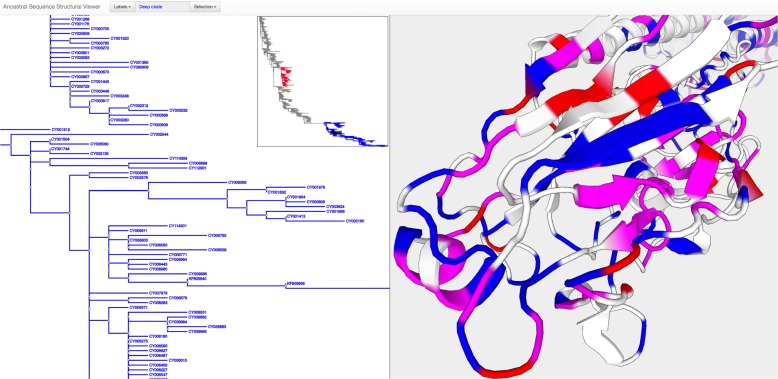
Interfacing with PV, the JavaScript protein viewer, to interactively view substitutions inferred by an evolutionary model. Both libraries are documented and provide useful abstractions, so that combining them into one interoperable application can be achieved with a few dozen lines of code

Our second example is used to illustrate how phylotree.js can be used to interface with existing software libraries. Figure 2 depicts a standalone application that is used to visualize amino-acid substitutions inferred by ancestral sequence reconstruction on a protein structure (Influenza A virus hemagglutinin). The alignment was taken from a molecular evolutionary study of this protein [14]. Ancestral amino-acid substitutions were inferred by SLAC [15]. Upon selecting a set of branches, any non-synonymous substitutions that occur within the selected set will be mapped to their position on the structure and automatically highlighted. Two different types of selection are permitted, shown in red and blue, permitting to contrast substitution patterns in different parts of the tree. The protein structure was visualized using the PV JavaScript library [16].

Since phylotree.js and PV are both documented and provide abstractions that are useful to software developers, it is straightforward to integrate them. Moreover, this example demonstrates the ability of phylotree.js to display large trees. The tree is too large to fit entirely in the browser’s window, so that instead users can scroll through and observe fine details of individual branches and their neighbors. The embedded, clickable “picture in picture” view shows the complete tree (with unintelligible details), with the user’s current location in the larger (zoomed in) tree.

**Fig. 3:**
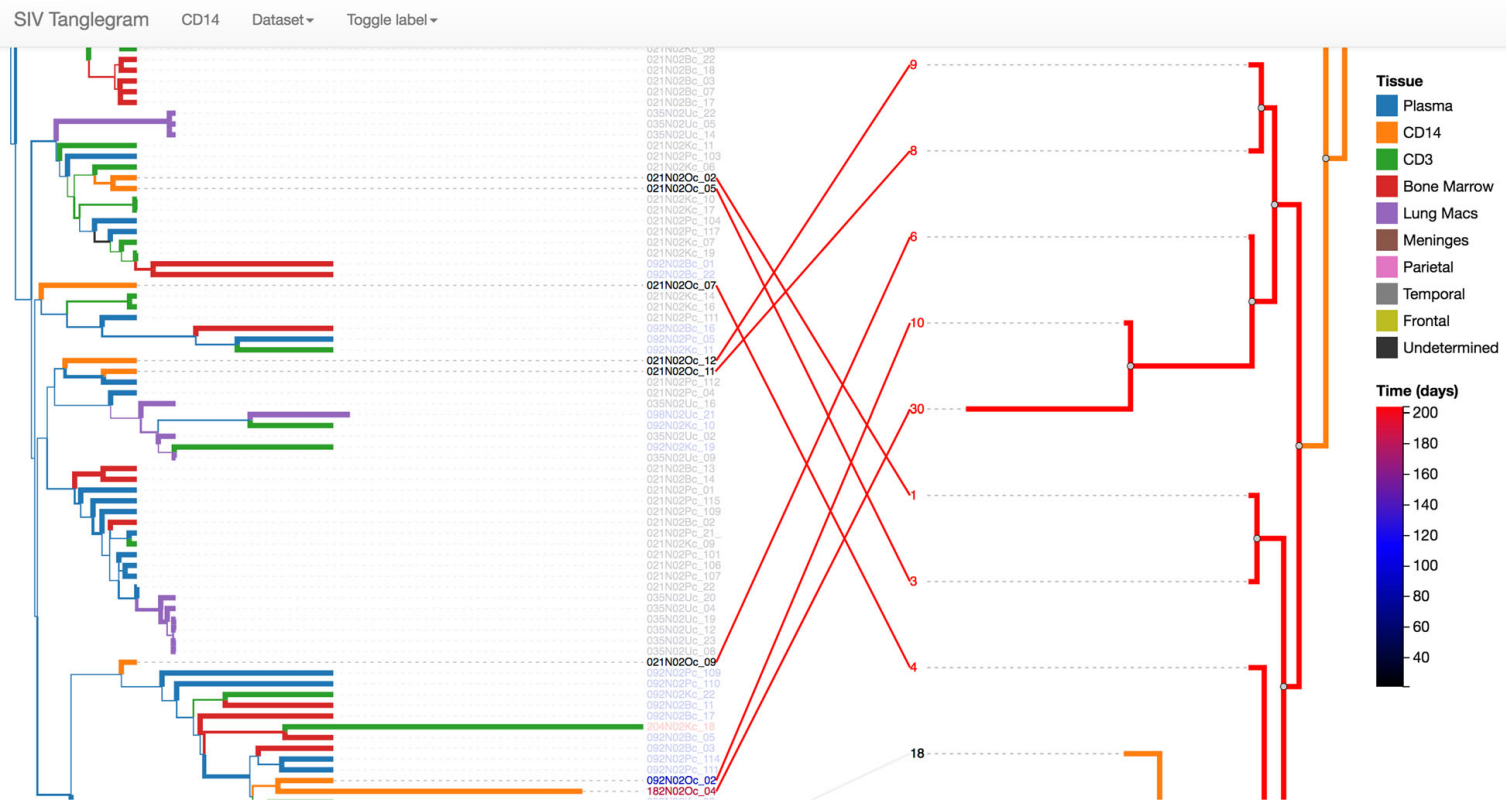
An interactive tanglegram of SIV sequencing data obtained from multiple tissues in primates. The single-tissue tree topology is compared with that of multiple tissues. Crossings can be selected and highlighted in the single tissue tree, and reducing the number of such crossings is necessary to avoid spurious biological conclusions. This is achieved by implementing a divide-and-conquer strategy using phylotree.js’s algorithmic abstractions

Finally, phylotree.js has been used to implement a side-by-side comparison of phylogenetic trees with links between leaves, otherwise known as a tanglegram (depicted in Fig. 3). Crossings can represent interesting evolutionary events, or highlight the disparity between a single vs. multi-tissue tree as in this example. However, the layout must be done with care, since the ordering of a node’s children does not affect the tree topology but can artificially inflate the number of crossings. It is straightforward to implement a version of the dynamic programming algorithm described in [17] to minimize crossings using the algorithmic abstractions that are provided.

## Conclusions

phylotree.js is licensed permissively under the MIT license, is well documented, and contains a gallery of examples. The code at time of writing is available as Additional file 1. A variety of distinct uses have been given, all based upon the notion of extensible branch-selection middleware. phylotree.js strives to be the appropriate tool for use in larger JavaScript applications that involve users selecting portions phylogenetic trees for downstream consumption in a manner that is not necessarily pre-defined, as well as a layout in D3 for custom, interactive data visualization.

## Availability and requirements

**Project name** phylotree.js

**Project home page** https://github.com/veg/phylotree.js

**Operating system(s)** Platform independent

**Programming language** JavaScript

**Other requirements** d3.js 3.x

**License** MIT

**Any restrictions to use by non-academics** None

## Additional file

Additional file 1 Latest release of source code. A zip file of the source code from release 0.1.8. Accessed 4 May 2018. (ZIP 3513 kb)

## Abbreviations

API: Application programming interface
D3: Data driven documents
PV: Protein viewer
SLAC: Single likelihood ancestor counting
XML: Extensible markup language

## References

[1] Darwin C (1859). On the Origin of Species by Means of Natural Selection, or, the Preservation of Favoured Races in the Struggle for Life.

[2] Vaughan TG (2017). IcyTree: rapid browser-based visualization for phylogenetic trees and networks. Bioinformatics.

[3] Kreft Ł, Botzki A, Coppens F, Vandepoele K, Van Bel M (2017). PhyD3: a phylogenetic tree viewer with extended phyloXML support for functional genomics data visualization. Bioinformatics.

[4] Robinson O, Dylus D, Dessimoz C (2016). Phylo. io: interactive viewing and comparison of large phylogenetic trees on the web. Mol Biol Evol.

[5] Smits SA, Ouverney CC (2010). jsPhyloSVG: a javascript library for visualizing interactive and vector-based phylogenetic trees on the web. PLoS ONE.

[6] Phylotree Web Application. http://phylotree.hyphy.org/. Accessed 17 Jan 2018.

[7] Bostock M, Ogievetsky V, Heer J (2011). D3 data-driven documents. IEEE Trans Vis Comput Graph.

[8] Pond SLK, Muse SV (2005). HyPhy: hypothesis testing using phylogenies. Statistical methods in molecular evolution.

[9] Drummond AJ (2007). BEAST: Bayesian evolutionary analysis by sampling trees. BMC Evol Biol.

[10] Han MV, Zmasek CM (2009). phyloXML: XML for evolutionary biology and comparative genomics. BMC Bioinformatics.

[11] Vos RA, Balhoff JP, Caravas JA, Holder MT, Lapp H, Maddison WP (2012). NeXML: rich, extensible, and verifiable representation of comparative data and metadata. Syst Biol.

[12] Weaver S, Shank SD, Spielman SJ, Li M, Muse SV, Kosakovsky PSL (2018). Datamonkey 2.0: a modern web application for characterizing selective and other evolutionary processes. Mol Biol Evol.

[13] Wertheim JO, Murrell B, Smith MD, Kosakovsky PSL, Scheffler K (2014). RELAX: detecting relaxed selection in a phylogenetic framework. Mol Biol Evol.

[14] Meyer AG, Wilke CO (2015). Geometric constraints dominate the antigenic evolution of influenza H3N2 hemagglutinin. PLoS Pathog.

[15] Kosakovsky PSL, Frost SDW (2005). Not So Different After All: A Comparison of Methods for Detecting Amino Acid Sites Under Selection. Mol Biol Evol.

[16] Biasini M. pv: v1.8.1. 2015. Available from: 10.5281/zenodo.20980.

[17] Venkatachalam B, Apple J, St John K, Gusfield D (2010). Untangling tanglegrams: Comparing trees by their drawings. IEEE/ACM Trans Comput Biol Bioinforma (TCBB).

