# Supplementary material for: phylotree.js - a JavaScript library for application development and interactive data visualization in phylogenetics
Source: BMC Bioinformatics. 2018 Jul 25;19:276. doi: 10.1186/s12859-018-2283-2 (PMC6060545; doi:10.1186/s12859-018-2283-2)
Supplement: Supplementary file 1 — Latest release of source code. A zip file of the source code from release 0.1.8. Accessed 4 May 2018. (ZIP 3513 kb) [file 12859_2018_2283_MOESM1_ESM.zip › phylotree.js-0.1.8/documentation/advanced.html]

  


Advanced — Phylotree.js 0.1.5 documentation


Phylotree.js

0.1.5

- Introduction
  - Installation
  - A minimal working example
  - Toggling options
- Fundamentals
  - Reading and writing trees
  - Drawing trees
  - Formatting trees
- Options
- Nodes and branches
  - Node methods
  - Branch methods
- Selection
- Advanced
- Examples

Phylotree.js

- Docs »
- Advanced
- View page source

---

# Advanced¶

This will describe advanced methods. These are methods that are often called internally
and are exposed for completeness, but do not make up typical use cases.

`phylotree.``placenodes`()¶
:   Place the current nodes, i.e., determine their coordinates based
    on current settings.

    |  |  |
    | --- | --- |
    | Returns: | The current `phylotree`. |

`phylotree.``update`(*transitions*)¶
:   Update the current phylotree, i.e., alter the svg
    elements.

    |  |  |
    | --- | --- |
    | Arguments: | - **transitions** (*Boolean*) – (Optional) Toggle whether transitions should be shown. |
    | Returns: | The current `phylotree`. |

Next 
 Previous

---

© Copyright 2017, VEG/IGEM.

Built with Sphinx using a theme provided by Read the Docs.
