# Supplementary material for: phylotree.js - a JavaScript library for application development and interactive data visualization in phylogenetics
Source: BMC Bioinformatics. 2018 Jul 25;19:276. doi: 10.1186/s12859-018-2283-2 (PMC6060545; doi:10.1186/s12859-018-2283-2)
Supplement: Supplementary file 1 — Latest release of source code. A zip file of the source code from release 0.1.8. Accessed 4 May 2018. (ZIP 3513 kb) [file 12859_2018_2283_MOESM1_ESM.zip › phylotree.js-0.1.8/documentation/examples.html]

  


Examples — Phylotree.js 0.1.5 documentation


Phylotree.js

0.1.5

- Introduction
  - Installation
  - A minimal working example
  - Toggling options
- Fundamentals
  - Reading and writing trees
  - Drawing trees
  - Formatting trees
- Options
- Nodes and branches
  - Node methods
  - Branch methods
- Selection
- Advanced
- Examples

Phylotree.js

- Docs »
- Examples
- View page source

---

# Examples¶

Here is a gallery of phylotree examples.

Hello-world
:   A working example with the minimal amount of coding.

    - View Hello-world example
    - View Hello-world source

Phylo-bar
:   A phylogenetic tree that interfaces with a D3.js bar chart to count classes of substitutions along portions of a phylogeny.

    - View Phylo-bar example
    - View Phylo-bar source

Vanilla
:   The simplest tree possible, with all default features disabled.

    - View Vanilla example
    - View Vanilla source

Custom menu items
:   Adding a custom menu item to italicize node names. Leaf node menus now have an additional option “Italicize node label”.

    - View Custom menu items example
    - View Custom menu items source

Radial tree
:   A tree with a button to toggle a radial layout.

    - View Radial tree example
    - View Radial tree source

Branch coloring and thickness
:   Adjust color of branches by membership value and thickness by bootstrap value.

    - View Branch coloring example
    - View Branch coloring source

Variable size and shape for leaf nodes
:   Draw different shapes and sizes for leaf nodes (based on haplotype diversity).

    - View Variable leaves example
    - View Variable leaves source

Zoom
:   Example of zooming and panning.

    - View Zooming example
    - View Zooming source

Leaf metadata
:   Visualize some artificial data associated with leaves of a tree.

    - View Leaf metadata example
    - View Leaf metadata source

XML parsing
:   Parse PhyloXML or NexML formats.

    - View PhyloXML example
    - View PhyloXML source
    - View NexML example
    - View NexML source

Large tree
:   Viewing a large tree with a Starcraft-style guide.

    - View Large tree example
    - View Large tree source

Web application
:   A web application for visualizing and annotating phylogenetic trees. Load trees as files or text, make multiple
    named selections with a variety of mechanisms, and save annotated trees for further use.

    - View Web application example
    - View Web application source

Ancestral sequence structural viewer
:   Interfacing with PV, the Javascript protein viewer, to visualize
    ancestral substitutions on a protein structure. HyPhy was used to infer ancestral substitutions on an alignment of hemagglutinin.
    Users can make two types of branch selections. Any branch that is selected and has inferred non-synonymous substitutions will
    instantaneously highlight positions where those substitutions occurred on an associated protein structure.

    - View Ancestral sequence structural viewer example
    - View Ancestral sequence structural viewer source

Interactive tanglegram
:   A tanglegram to compare phylogenies inferred from muliple vs. single tissue samples. Crossings are minimized using a
    dynamic programming algorithm, and the single-tissue tree enables selections to highlight inter-tree crossings.

    - View Interactive tanglegram example
    - View Interactive tanglegram source

Previous

---

© Copyright 2017, VEG/IGEM.

Built with Sphinx using a theme provided by Read the Docs.
