# Supplementary material for: phylotree.js - a JavaScript library for application development and interactive data visualization in phylogenetics
Source: BMC Bioinformatics. 2018 Jul 25;19:276. doi: 10.1186/s12859-018-2283-2 (PMC6060545; doi:10.1186/s12859-018-2283-2)
Supplement: Supplementary file 1 — Latest release of source code. A zip file of the source code from release 0.1.8. Accessed 4 May 2018. (ZIP 3513 kb) [file 12859_2018_2283_MOESM1_ESM.zip › phylotree.js-0.1.8/documentation/fundamentals.html]

  


Fundamentals — Phylotree.js 0.1.5 documentation


Phylotree.js

0.1.5

- Introduction
  - Installation
  - A minimal working example
  - Toggling options
- Fundamentals
  - Reading and writing trees
  - Drawing trees
  - Formatting trees
- Options
- Nodes and branches
  - Node methods
  - Branch methods
- Selection
- Advanced
- Examples

Phylotree.js

- Docs »
- Fundamentals
- View page source

---

# Fundamentals¶

This section describes basic commands for displaying trees, such as those found in
the Introduction section.

Note that many methods follow the getter/setter pattern, commonly used in D3. That is,
they can either be used to retrieve an underlying parameter by being invoked without
arguments (get), or can be used to change an underlying parameter by being invoked with the
proper arguments (set).

## Reading and writing trees¶

The following is the main method for instantiating a phylotree. It is
attached to the `d3.layout` namespace.

`d3.layout.``phylotree`([*container*])¶
:   Instantiate a phylotree.

    |  |  |
    | --- | --- |
    | Arguments: | - **container** (*d3-selection*) – Specify a container, for things like menu and tooltip placement. Defaults to body (optional). |
    | Returns: | **function** – phylotree - an instance of a Phylotree. |

Phylotrees are themselves functions, with many methods attached and variables
that have been closed over to provide an internal state. Newick, PhyloXML, and
NeXML formats are supported.

`phylotree.``phylotree`(*nwk*[, *bootstrap\_values*])¶
:   An instance of a phylotree. Sets event listeners, parses tags, and creates links
    that represent branches.

    |  |  |
    | --- | --- |
    | Arguments: | - **nwk** (*Object*) – A Newick string, PhyloXML string, or hierarchical JSON representation of a phylogenetic tree. - **bootstrap\_values** (*Object*) – SDS: Not sure what this does. |
    | Returns: | **Phylotree** – phylotree - itself, following the builder pattern. |

`d3.layout.phylotree.``nexml_parser`(*xml\_string*)¶
:   A parser for NexML. This is a separate function, since NeXML objects
    can contain multiple trees. Results should be passed into a phylotree
    object, as shown in the examples.

    |  |  |
    | --- | --- |
    | Arguments: | - **nexml** (*Object*) – A NeXML string. |
    | Returns: | **Object** – trees - An array of trees contained in the NeXML object. |

Internally, Phylotree.js uses the D3 hierarchy layout. The following function
parses Newick strings into a hierarchical JSON format. Certain ad-hoc extensions,
such as those used by HyPhy or Beast, are (partially) supported.

`d3.layout.``newick_parser`(*nwk\_str*[, *bootstrap\_values*])¶
:   Parses a Newick string into an equivalent JSON representation that is
    suitable for consumption by `d3.layout.hierarchy`.

    Optionally accepts bootstrap values. Currently supports Newick strings with or without branch lengths,
    as well as tagged trees such as

    > `(a,(b{TAG},(c{TAG},d{ANOTHERTAG})))`

    |  |  |
    | --- | --- |
    | Arguments: | - **nwk\_str** (*String*) – A string representing a phylogenetic tree in Newick format. - **bootstrap\_values** (*Object*) – SDS: Not clear what this does (optional). |
    | Returns: | **Object** – An object with keys `json` and `error`. |

Trees may be serialized to Newick strings, possibly after having been annotated,
for downstream use in an application.

`phylotree.``get_newick`([*annotator*])¶
:   Return Newick string representation of a phylotree.

    |  |  |
    | --- | --- |
    | Arguments: | - **annotator** (*function*) – Function to apply to each node, determining what label is written (optional). |
    | Returns: | **String** – newick - Phylogenetic tree serialized as a Newick string. |

`phylotree.``get_parsed_tags`()¶
:   Return tags that were read when parsing the original Newick string.

    |  |  |
    | --- | --- |
    | Returns: | An array of strings, comprising each tag that was read. |

## Drawing trees¶

In order to render a phylotree, an SVG element needs to present in the body of the document. One
can specify which svg to render within with the following function.

`phylotree.``svg`(*svg\_element*)¶
:   Getter/setter for the SVG element for the Phylotree to be rendered in.

    |  |  |
    | --- | --- |
    | Arguments: | - **svg\_element** (*d3-selection*) – (Optional) SVG element to render within, selected by D3. |
    | Returns: | The selected SVG element if getting, or the current `phylotree` if setting.` |

Once a tree has been parsed and an SVG element chosen, the following function is called to render
the tree.

`phylotree.``layout`([*transitions*])¶
:   Lay out the tree within the SVG.

    |  |  |
    | --- | --- |
    | Arguments: | - **transitions** (*Boolean*) – Specify whether or not transitions should occur. |
    | Returns: | The current `phylotree`. |

## Formatting trees¶

The following methods are getters/setters for various formatting options.

`phylotree.``size`([*attr*])¶
:   Get or set the size of tree in pixels.

    |  |  |
    | --- | --- |
    | Arguments: | - **attr** (*Array*) – (optional) An array of the form `[height, width]`. |
    | Returns: | **Phylotree** – The current `size` array if getting, or the current `phylotree` if setting. |

`phylotree.``spacing_x`([*attr*, *skip\_render*])¶
:   Get or set spacing in the x-direction.

    |  |  |
    | --- | --- |
    | Arguments: | - **attr** (*Number*) – (Optional), the new spacing value if setting. - **skip\_render** (*Boolean*) – (Optional), whether or not a refresh should be performed. |
    | Returns: | The current `spacing_x` value if getting, or the current `phylotree` if setting. |

`phylotree.``spacing_y`([*attr*, *skip\_render*])¶
:   Get or set spacing in the y-direction.

    |  |  |
    | --- | --- |
    | Arguments: | - **attr** (*Number*) – (Optional), the new spacing value if setting. - **skip\_render** (*Boolean*) – (Optional), whether or not a refresh should be performed. |
    | Returns: | The current `spacing_y` value if getting, or the current `phylotree` if setting. |

`phylotree.``font_size`([*attr*])¶
:   Get or set font size.

    |  |  |
    | --- | --- |
    | Arguments: | - **attr** (*function*) – Empty if getting, or new font size if setting. |
    | Returns: | The current `font_size` accessor if getting, or the current `phylotree` if setting. |

Next 
 Previous

---

© Copyright 2017, VEG/IGEM.

Built with Sphinx using a theme provided by Read the Docs.
