# Supplementary material for: phylotree.js - a JavaScript library for application development and interactive data visualization in phylogenetics
Source: BMC Bioinformatics. 2018 Jul 25;19:276. doi: 10.1186/s12859-018-2283-2 (PMC6060545; doi:10.1186/s12859-018-2283-2)
Supplement: Supplementary file 1 — Latest release of source code. A zip file of the source code from release 0.1.8. Accessed 4 May 2018. (ZIP 3513 kb) [file 12859_2018_2283_MOESM1_ESM.zip › phylotree.js-0.1.8/documentation/genindex.html]

  


Index — Phylotree.js 0.1.5 documentation


Phylotree.js

0.1.5

- Introduction
  - Installation
  - A minimal working example
  - Toggling options
- Fundamentals
  - Reading and writing trees
  - Drawing trees
  - Formatting trees
- Options
- Nodes and branches
  - Node methods
  - Branch methods
- Selection
- Advanced
- Examples

Phylotree.js

- Docs »
- Index

---

# Index

**D**
| **P**

## D

|  |  |
| --- | --- |
| - d3.layout.newick\_parser() (d3.layout method) | - d3.layout.phylotree() (d3.layout method) - d3.layout.phylotree.nexml\_parser() (d3.layout.phylotree method) |

## P

|  |  |
| --- | --- |
| - phylotree.branch\_length() (phylotree method) - phylotree.collapse\_node() (phylotree method) - phylotree.delete\_a\_node() (phylotree method) - phylotree.descendants() (phylotree method) - phylotree.font\_size() (phylotree method) - phylotree.get\_newick() (phylotree method) - phylotree.get\_nodes() (phylotree method) - phylotree.get\_parsed\_tags() (phylotree method) - phylotree.get\_selection() (phylotree method) - phylotree.layout() (phylotree method) - phylotree.modify\_selection() (phylotree method) - phylotree.node\_span() (phylotree method) - phylotree.options() (phylotree method) - phylotree.phylotree() (phylotree method) | - phylotree.placenodes() (phylotree method) - phylotree.reroot() (phylotree method) - phylotree.select\_all\_descendants() (phylotree method) - phylotree.selection\_callback() (phylotree method) - phylotree.selection\_label() (phylotree method) - phylotree.size() (phylotree method) - phylotree.spacing\_x() (phylotree method) - phylotree.spacing\_y() (phylotree method) - phylotree.style\_edges() (phylotree method) - phylotree.style\_nodes() (phylotree method) - phylotree.svg() (phylotree method) - phylotree.toggle\_collapse() (phylotree method) - phylotree.traverse\_and\_compute() (phylotree method) - phylotree.update() (phylotree method) |

---

© Copyright 2017, VEG/IGEM.

Built with Sphinx using a theme provided by Read the Docs.
