# Supplementary material for: phylotree.js - a JavaScript library for application development and interactive data visualization in phylogenetics
Source: BMC Bioinformatics. 2018 Jul 25;19:276. doi: 10.1186/s12859-018-2283-2 (PMC6060545; doi:10.1186/s12859-018-2283-2)
Supplement: Supplementary file 1 — Latest release of source code. A zip file of the source code from release 0.1.8. Accessed 4 May 2018. (ZIP 3513 kb) [file 12859_2018_2283_MOESM1_ESM.zip › phylotree.js-0.1.8/documentation/index.html]

  


Welcome to Phylotree.js’s documentation! — Phylotree.js 0.1.5 documentation


Phylotree.js

0.1.5

- Introduction
  - Installation
  - A minimal working example
  - Toggling options
- Fundamentals
  - Reading and writing trees
  - Drawing trees
  - Formatting trees
- Options
- Nodes and branches
  - Node methods
  - Branch methods
- Selection
- Advanced
- Examples

Phylotree.js

- Docs »
- Welcome to Phylotree.js’s documentation!
- View page source

---

# Welcome to Phylotree.js’s documentation!¶

Phylotree.js is a Javascript library for working with phylogenetic trees. Uses include web application development and interactive data visualization for phylogenetics.

It is written as a D3 layout, and aspires to be a general purpose tool for visualizing and selecting branches in phylogenetic trees. Example uses include a standalone web application for viewing and labeling phylogenetic trees, the Datamonkey webserver for comparative sequence analysis, an ancestral sequence structural viewer, and an interactive tanglegram.

The code is available on Github.

## Contents¶

- Introduction
  - Installation
  - A minimal working example
  - Toggling options
- Fundamentals
  - Reading and writing trees
  - Drawing trees
  - Formatting trees
- Options
- Nodes and branches
  - Node methods
  - Branch methods
- Selection
- Advanced
- Examples

## Indices and tables¶

- Index
- Search Page

Next

---

© Copyright 2017, VEG/IGEM.

Built with Sphinx using a theme provided by Read the Docs.
