# Supplementary material for: phylotree.js - a JavaScript library for application development and interactive data visualization in phylogenetics
Source: BMC Bioinformatics. 2018 Jul 25;19:276. doi: 10.1186/s12859-018-2283-2 (PMC6060545; doi:10.1186/s12859-018-2283-2)
Supplement: Supplementary file 1 — Latest release of source code. A zip file of the source code from release 0.1.8. Accessed 4 May 2018. (ZIP 3513 kb) [file 12859_2018_2283_MOESM1_ESM.zip › phylotree.js-0.1.8/documentation/intro.html]

  


Introduction — Phylotree.js 0.1.5 documentation


Phylotree.js

0.1.5

- Introduction
  - Installation
  - A minimal working example
  - Toggling options
- Fundamentals
  - Reading and writing trees
  - Drawing trees
  - Formatting trees
- Options
- Nodes and branches
  - Node methods
  - Branch methods
- Selection
- Advanced
- Examples

Phylotree.js

- Docs »
- Introduction
- View page source

---

# Introduction¶

Getting started with Phylotree.js is straightforward. This page describes acquiring the source code and two simple examples for displaying trees on a web page.

## Installation¶

The source code for Phylotree is available as a single Javascript file. An additional CSS file is provided for appropriate SVG styling. Phylotree
also requires D3, jQuery, Underscore, and Bootstrap as dependencies. To use Phylotree.js, make sure all of Phylotree and its dependencies are included
in your project.

If you are developing web applications in the traditional manner (i.e., by including Javascript and CSS files in HTML files), acquire the source code at Github by
downloading a compressed version of the latest release. Then, include the Javascript and CSS as usual:

```
<link rel="stylesheet" href="//netdna.bootstrapcdn.com/bootstrap/3.3.5/css/bootstrap.min.css">
<link rel="stylesheet" href="//netdna.bootstrapcdn.com/bootstrap/3.3.5/css/bootstrap-theme.min.css">
<link rel="stylesheet" href="phylotree.css">

<script src="//code.jquery.com/jquery.js"></script>
<script src="//netdna.bootstrapcdn.com/bootstrap/3.3.5/js/bootstrap.min.js"></script>
<script src="//d3js.org/d3.v3.min.js"></script>
<script src="https://cdnjs.cloudflare.com/ajax/libs/underscore.js/1.8.3/underscore-min.js" charset="utf-8"></script>
<script type='text/javascript' src='phylotree.js'></script>
```

Phylotree.js is also available as an NPM package. It can be imported as an ES6 module and bundled with a module-bundler like Webpack. Install with NPM via

```
npm install phylotree
```

or yarn via

```
yarn add phylotree
```

to obtain the source code. Then include the necessary Javascript and CSS using the following ES6 syntax:

```
require("phylotree");
require("phylotree.css");
```

## A minimal working example¶

If you have a tree in Newick format, you are ready to go with the following four lines of Javascript. Below,
we assume such a string to be stored in the variable `example_tree`.

```
var tree = d3.layout.phylotree()
  .svg(d3.select("#tree_display"));
tree(example_tree)
  .layout();
```

This forms the basis of the Hello World example.

## Toggling options¶

The above example comes complete with selectable branches, nodes with drop down menus, clades that collapse, and
so on. These can be toggled with the options method. The following code snippet simply displays a tree, disabling
the default options.

```
var height = 400,
  width = 400,

  tree = d3.layout.phylotree()
  .svg(d3.select("#tree_display"))
  .options({
    'left-right-spacing': 'fit-to-size',
    // fit to given size top-to-bottom
    'top-bottom-spacing': 'fit-to-size',
    // fit to given size left-to-right
    'selectable': false,
    // make nodes and branches not selectable
    'collapsible': false,
    // turn off the menu on internal nodes
    'transitions': false
    // turn off d3 animations
  })
  .size([height, width])
  .node_circle_size(0); // do not show "circles" at internal nodes

tree(newick)
  // generate node coordinates
  .layout();
```

This forms the basis of the Vanilla example. A list of available options is available in the Options section.

Next 
 Previous

---

© Copyright 2017, VEG/IGEM.

Built with Sphinx using a theme provided by Read the Docs.
