# Supplementary material for: phylotree.js - a JavaScript library for application development and interactive data visualization in phylogenetics
Source: BMC Bioinformatics. 2018 Jul 25;19:276. doi: 10.1186/s12859-018-2283-2 (PMC6060545; doi:10.1186/s12859-018-2283-2)
Supplement: Supplementary file 1 — Latest release of source code. A zip file of the source code from release 0.1.8. Accessed 4 May 2018. (ZIP 3513 kb) [file 12859_2018_2283_MOESM1_ESM.zip › phylotree.js-0.1.8/documentation/nodes_branches.html]

  


Nodes and branches — Phylotree.js 0.1.5 documentation


Phylotree.js

0.1.5

- Introduction
  - Installation
  - A minimal working example
  - Toggling options
- Fundamentals
  - Reading and writing trees
  - Drawing trees
  - Formatting trees
- Options
- Nodes and branches
  - Node methods
  - Branch methods
- Selection
- Advanced
- Examples

Phylotree.js

- Docs »
- Nodes and branches
- View page source

---

# Nodes and branches¶

This will describe node- and branch-based methods.

## Node methods¶

`phylotree.``get_nodes`()¶
:   Get an array of all nodes.

    |  |  |
    | --- | --- |
    | Returns: | **Array** – Nodes in the current `phylotree`. |

`phylotree.``descendants`(*n*)¶
:   Get all descendants of a given node.

    |  |  |
    | --- | --- |
    | Arguments: | - **node** (*Node*) – A node in the current phylotree. |
    | Returns: | **Array** – An array of descendant nodes. |

`phylotree.``collapse_node`(*n*)¶
:   Collapses a given node.

    |  |  |
    | --- | --- |
    | Arguments: | - **node** (*Node*) – A node to be collapsed. |

`phylotree.``style_nodes`([*attr*])¶
:   Get or set node styler. If setting, pass a function of two arguments,
    `element` and `data`. `data` exposes the underlying node so that
    its attributes can be referenced. These can be used to apply styles to
    `element`, which will be a D3 selection corresponding to the SVG element
    that makes up the current node.

    |  |  |
    | --- | --- |
    | Arguments: | - **attr** (*function*) – Optional; if setting, the node styler function to be set. |
    | Returns: | The `node_styler` function if getting, or the current `phylotree` if setting. |

`phylotree.``node_span`([*attr*])¶
:   Get or set the current node span. If setting, the argument should
    be a function of a node which returns a number, so that node spans
    can be determined dynamically. Alternatively, the argument can be the
    string `"equal"`, to give all nodes an equal span.

    |  |  |
    | --- | --- |
    | Arguments: | - **attr** (*function*) – Optional; if setting, the node\_span function. |
    | Returns: | The `node_span` if getting, or the current `phylotree` if setting. |

`phylotree.``delete_a_node`(*index*)¶
:   Delete a given node.

    |  |  |
    | --- | --- |
    | Arguments: | - **The** (*Node*) – node to be deleted, or the index of such a node. |
    | Returns: | The current `phylotree`. |

`phylotree.``traverse_and_compute`(*callback*, *traversal\_type*)¶
:   Traverse the tree in a prescribed order, and compute a value at each node.

    |  |  |
    | --- | --- |
    | Arguments: | - **callback** (*function*) – A function to be called on each node. - **traversal\_type** (*String*) – Either `"pre-order"` or `"post-order"`. |

`phylotree.``reroot`(*node*)¶
:   Reroot the tree on the given node.

    |  |  |
    | --- | --- |
    | Arguments: | - **node** (*Node*) – Node to reroot on. |
    | Returns: | **Phylotree** – The current `phylotree`. |

`phylotree.``toggle_collapse`(*node*)¶
:   Toggle collapsed view of a given node. Either collapses a clade into
    a smaller blob for viewing large trees, or expands a node that was
    previously collapsed.

    |  |  |
    | --- | --- |
    | Arguments: | - **node** (*Node*) – The node to toggle. |
    | Returns: | **Phylotree** – The current `phylotree`. |

## Branch methods¶

`phylotree.``style_edges`([*attr*])¶
:   Get or set edge styler. If setting, pass a function of two arguments,
    `element` and `data`. `data` exposes the underlying edge so that
    its attributes can be referenced. These can be used to apply styles to
    `element`, which will be a D3 selection corresponding to the SVG element
    that makes up the current edge.

    Note that, in accordance with the D3 hierarchy layout, edges will have
    a `source` and `target` field, corresponding to the nodes that make up
    up the associated branch.

    |  |  |
    | --- | --- |
    | Arguments: | - **attr** (*function*) – Optional; if setting, the node styler function to be set. |
    | Returns: | The `edge_styler` function if getting, or the current `phylotree` if setting. |

`phylotree.``branch_length`([*attr*])¶
:   Get or set branch length accessor.

    |  |  |
    | --- | --- |
    | Arguments: | - **attr** (*function*) – Empty if getting, or new branch length accessor if setting. |
    | Returns: | **Object** – The branch length accessor if getting, or the current phylotree if setting. |

Next 
 Previous

---

© Copyright 2017, VEG/IGEM.

Built with Sphinx using a theme provided by Read the Docs.
