# Supplementary material for: phylotree.js - a JavaScript library for application development and interactive data visualization in phylogenetics
Source: BMC Bioinformatics. 2018 Jul 25;19:276. doi: 10.1186/s12859-018-2283-2 (PMC6060545; doi:10.1186/s12859-018-2283-2)
Supplement: Supplementary file 1 — Latest release of source code. A zip file of the source code from release 0.1.8. Accessed 4 May 2018. (ZIP 3513 kb) [file 12859_2018_2283_MOESM1_ESM.zip › phylotree.js-0.1.8/documentation/options.html]

  


Options — Phylotree.js 0.1.5 documentation


Phylotree.js

0.1.5

- Introduction
  - Installation
  - A minimal working example
  - Toggling options
- Fundamentals
  - Reading and writing trees
  - Drawing trees
  - Formatting trees
- Options
- Nodes and branches
  - Node methods
  - Branch methods
- Selection
- Advanced
- Examples

Phylotree.js

- Docs »
- Options
- View page source

---

# Options¶

phylotree.js supports a variety of options for common features, which can be set using
the following function.

`phylotree.``options`(*opt*[, *run\_update*])¶
:   Change option settings.

    |  |  |
    | --- | --- |
    | Arguments: | - **opt** (*Object*) – Keys are the option to toggle and values are the parameters for that option. - **run\_update** (*Boolean*) – (optional) Whether or not the tree should update. |
    | Returns: | The current `phylotree`. |

The following are a list of possible options, along with their types, meanings, and possible values.

left-right-spacing
:   (String) Determines layout size from left to right. Defaults to `"fixed-step"`.

    - `"fixed-step"` - Determine width from padding and spacing.
    - `"fit-to-size"` - Determine width from size array.

top-bottom-spacing
:   (String) Determines layout size from top to bottom. Defaults to `"fixed-step"`.

    - `"fixed-step"` - Determine width from padding and spacing.
    - `"fit-to-size"` - Determine width from size array.

brush
:   (Boolean) Whether or not the brush should be activated. Defaults to `true`.

hide
:   (Boolean) Whether or not hiding a given node or subtree is enabled. Defaults to `true`.

reroot
:   (Boolean) Whether or not rerooting on a given node is enabled. Defaults to `true`.

compression
:   (Number) The percentage of original size for a collapsed node. Defaults to `.2`.

show-scale
:   (Boolean) Determines whether or not scale bar for branch lengths is shown.

left-offset
:   (Number) Amount of space on left side of phylotree. Defaults to `0`.

draw-size-bubbles
:   (Boolean) Determines whether nodes are drawn with a given size. Defaults to `false`.

max-radius
:   (Number) Set an upper bound on the radius in a radial layout. Defaults to 768.

collapsible
:   (Boolean) Determines whether or not nodes are collapsible. Defaults to `true`.

selectable
:   (Boolean) Determines whether or not individual branches are selectable. Defaults to `true`.

zoom
:   (Boolean) Determines whether or not zooming is enabled. Defaults to `false`.

restricted-selectable
:   (Array) Determines what types of global selection actions are possible. Defaults to `false`.

    - `false` - No restrictions placed on global selection.
    - `"all"` - Allow users to select all branches.
    - `"none"` - Allow users to unselect all branches.
    - `"all-leaf-nodes"` - Allow users to select all leaf nodes.
    - `"all-internal-branches"` - Allow users to select all internal branches.

align-tips
:   (Boolean) Determines whether tip names are aligned or not. Defaults to false.

maximum-per-node-spacing
:   (Number) Determines maximum node spacing allocated when laying out left to right. Defaults to 100.

minimum-per-node-spacing
:   (Number) Determines minimum node spacing allocated when laying out left to right. Defaults to 2.

maximum-per-level-spacing
:   (Number) Determines maximum node spacing allocated when laying out top to bottom. Defaults to 100.

minimum-per-level-spacing
:   (Number) Determines minimum node spacing allocated when laying out top to bottom. Defaults to 10.

Next 
 Previous

---

© Copyright 2017, VEG/IGEM.

Built with Sphinx using a theme provided by Read the Docs.
