# Supplementary material for: phylotree.js - a JavaScript library for application development and interactive data visualization in phylogenetics
Source: BMC Bioinformatics. 2018 Jul 25;19:276. doi: 10.1186/s12859-018-2283-2 (PMC6060545; doi:10.1186/s12859-018-2283-2)
Supplement: Supplementary file 1 — Latest release of source code. A zip file of the source code from release 0.1.8. Accessed 4 May 2018. (ZIP 3513 kb) [file 12859_2018_2283_MOESM1_ESM.zip › phylotree.js-0.1.8/documentation/selection.html]

  


Selection — Phylotree.js 0.1.5 documentation


Phylotree.js

0.1.5

- Introduction
  - Installation
  - A minimal working example
  - Toggling options
- Fundamentals
  - Reading and writing trees
  - Drawing trees
  - Formatting trees
- Options
- Nodes and branches
  - Node methods
  - Branch methods
- Selection
- Advanced
- Examples

Phylotree.js

- Docs »
- Selection
- View page source

---

# Selection¶

This will describe methods that pertain to making selections.

`phylotree.``modify_selection`(*node\_selecter*, *attr*, *place*, *skip\_refresh*, *mode*)¶
:   Modify the current selection, via functional programming.

    |  |  |
    | --- | --- |
    | Arguments: | - **node\_selecter** (*function*) – A function to apply to each node, which determines whether they become part of the current selection. Alternatively, if `restricted-selectable` mode is enabled, a string describing one of the pre-defined restricted-selectable options. - **attr** (*String*) – (Optional) The selection attribute to modify. - **place** (*Boolean*) – (Optional) Whether or not `placenodes` should be called. - **skip\_refresh** (*Boolean*) – (Optional) Whether or not a refresh is called. - **mode** (*String*) – (Optional) Can be `"toggle"`, `"true"`, or `"false"`. |
    | Returns: | The current `phylotree`. |

`phylotree.``selection_callback`(*callback*)¶
:   Getter/setter for the selection callback. This function is called
    every time the current selection is modified, and its argument is
    an array of nodes that make up the current selection.

    |  |  |
    | --- | --- |
    | Arguments: | - **callback** (*function*) – (Optional) The selection callback function. |
    | Returns: | The current `selection_callback` if getting, or the current `phylotree` if setting. |

`phylotree.``selection_label`(*attr*)¶
:   Getter/setter for the selection label. Useful when allowing
    users to make multiple selections.

    |  |  |
    | --- | --- |
    | Arguments: | - **attr** (*String*) – (Optional) If setting, the new selection label. |
    | Returns: | The current selection label if getting, or the current `phylotree` if setting. |

`phylotree.``get_selection`()¶
:   Get nodes which are currently selected.

    |  |  |
    | --- | --- |
    | Returns: | **Array** – An array of nodes that match the current selection. |

`phylotree.``select_all_descendants`(*node*, *terminal*, *internal*)¶
:   Select all descendents of a given node, with options for selecting
    terminal/internal nodes.

    |  |  |
    | --- | --- |
    | Arguments: | - **node** (*Node*) – The node whose descendents should be selected. - **terminal** (*Boolean*) – Whether to include terminal nodes. - **internal** (*Boolean*) – Whther to include internal nodes. |
    | Returns: | **Array** – An array of selected nodes. |

Next 
 Previous

---

© Copyright 2017, VEG/IGEM.

Built with Sphinx using a theme provided by Read the Docs.
