# Supplementary material for: phylotree.js - a JavaScript library for application development and interactive data visualization in phylogenetics
Source: BMC Bioinformatics. 2018 Jul 25;19:276. doi: 10.1186/s12859-018-2283-2 (PMC6060545; doi:10.1186/s12859-018-2283-2)
Supplement: Supplementary file 1 — Latest release of source code. A zip file of the source code from release 0.1.8. Accessed 4 May 2018. (ZIP 3513 kb) [file 12859_2018_2283_MOESM1_ESM.zip › phylotree.js-0.1.8/examples/color-branches/index.html]

Radial layout 

Input Newick
Input clustering

×

#### Newick string to render

(a : 0.1, (b : 0.11, (c : 0.12, d : 0.13) : 0.14) : 0.15)

Display this tree

×

#### Clustering CSV

Seq1,Seq2,Seq3
A,B,A

Use this labeling
