# Supplementary material for: phylotree.js - a JavaScript library for application development and interactive data visualization in phylogenetics
Source: BMC Bioinformatics. 2018 Jul 25;19:276. doi: 10.1186/s12859-018-2283-2 (PMC6060545; doi:10.1186/s12859-018-2283-2)
Supplement: Supplementary file 1 — Latest release of source code. A zip file of the source code from release 0.1.8. Accessed 4 May 2018. (ZIP 3513 kb) [file 12859_2018_2283_MOESM1_ESM.zip › phylotree.js-0.1.8/examples/custom-menu-items/index.html]

```
var example_tree = "(((EELA:0.150276,CONGERA:0.213019):0.230956,(EELB:0.263487,CONGERB:0.202633):0.246917):0.094785,((CAVEFISH:0.451027,(GOLDFISH:0.340495,ZEBRAFISH:0.390163):0.220565):0.067778,((((((NSAM:0.008113,NARG:0.014065):0.052991,SPUN:0.061003,(SMIC:0.027806,SDIA:0.015298,SXAN:0.046873):0.046977):0.009822,(NAUR:0.081298,(SSPI:0.023876,STIE:0.013652):0.058179):0.091775):0.073346,(MVIO:0.012271,MBER:0.039798):0.178835):0.147992,((BFNKILLIFISH:0.317455,(ONIL:0.029217,XCAU:0.084388):0.201166):0.055908,THORNYHEAD:0.252481):0.061905):0.157214,LAMPFISH:0.717196,((SCABBARDA:0.189684,SCABBARDB:0.362015):0.282263,((VIPERFISH:0.318217,BLACKDRAGON:0.109912):0.123642,LOOSEJAW:0.397100):0.287152):0.140663):0.206729):0.222485,(COELACANTH:0.558103,((CLAWEDFROG:0.441842,SALAMANDER:0.299607):0.135307,((CHAMELEON:0.771665,((PIGEON:0.150909,CHICKEN:0.172733):0.082163,ZEBRAFINCH:0.099172):0.272338):0.014055,((BOVINE:0.167569,DOLPHIN:0.157450):0.104783,ELEPHANT:0.166557):0.367205):0.050892):0.114731):0.295021)"
// tree from Yokoyama et al http://www.ncbi.nlm.nih.gov/pubmed/18768804


var tree = d3.layout.phylotree()
  // create a tree layout object
  .svg(d3.select("#tree_display"));
  // render to this SVG element

function my_node_style_text(node) {
  node['text-italic'] = !node['text-italic'];
  d3.layout.phylotree.trigger_refresh(tree);
}

function my_menu_title(node) {
  if (node['text-italic']) {
    return "Remove Italics";
  }
  return "Italicize node label";
}

function my_style_nodes(element, node) {
  element.style("font-style", node['text-italic'] ? "italic" : "normal");
}
tree(d3.layout.newick_parser(example_tree))
  // parse the Newick into a d3 hierarchy object with additional fields
  .style_nodes(my_style_nodes)
  // handle custom node styling
  .layout();
  // layout and render the tree

// add a custom menu for (in this case) terminal nodes

tree.get_nodes().forEach(function(tree_node) {
  d3.layout.phylotree.add_custom_menu(tree_node, // add to this node
    my_menu_title, // display this text for the menu
    function() {
      my_node_style_text(tree_node);
    },
    // on-click callback include a reference to tree_node via transitive closure
    d3.layout.phylotree.is_leafnode // condition on when to display the menu
    // a function that takes node as an argument
  );
});
```
