# Supplementary material for: phylotree.js - a JavaScript library for application development and interactive data visualization in phylogenetics
Source: BMC Bioinformatics. 2018 Jul 25;19:276. doi: 10.1186/s12859-018-2283-2 (PMC6060545; doi:10.1186/s12859-018-2283-2)
Supplement: Supplementary file 1 — Latest release of source code. A zip file of the source code from release 0.1.8. Accessed 4 May 2018. (ZIP 3513 kb) [file 12859_2018_2283_MOESM1_ESM.zip › phylotree.js-0.1.8/examples/large-ancestral-structural-viewer/index.html]

Ancestral Sequence Structural Viewer


Toggle navigation

Ancestral Sequence Structural Viewer

Labels 

- Red selection
- Blue selection
- Rename selection


Cancel

Save

Selection 

- Select all
- Select all internal nodes
- Select all leaf nodes
- Clear all internal nodes
- Clear all leaves
- Clear selection
- Label internal nodes using maximum parsimony
- Label internal nodes using conjunction (AND)
- Label internal nodes using disjunction (OR)
