# Supplementary material for: phylotree.js - a JavaScript library for application development and interactive data visualization in phylogenetics
Source: BMC Bioinformatics. 2018 Jul 25;19:276. doi: 10.1186/s12859-018-2283-2 (PMC6060545; doi:10.1186/s12859-018-2283-2)
Supplement: Supplementary file 1 — Latest release of source code. A zip file of the source code from release 0.1.8. Accessed 4 May 2018. (ZIP 3513 kb) [file 12859_2018_2283_MOESM1_ESM.zip › phylotree.js-0.1.8/examples/radial/index.html]

Radial layout

```
d3.text("yokoyama.nwk", function(error, newick) {
  var tree = d3.layout.phylotree()
    .svg(d3.select("#tree_display"))
    .radial(true);

  tree(d3.layout.newick_parser(newick))
    .layout();

  $("#layout").on("click", function(e) {
    tree.radial($(this).prop("checked")).placenodes().update();
  });
});
```
