# Supplementary material for: phylotree.js - a JavaScript library for application development and interactive data visualization in phylogenetics
Source: BMC Bioinformatics. 2018 Jul 25;19:276. doi: 10.1186/s12859-018-2283-2 (PMC6060545; doi:10.1186/s12859-018-2283-2)
Supplement: Supplementary file 1 — Latest release of source code. A zip file of the source code from release 0.1.8. Accessed 4 May 2018. (ZIP 3513 kb) [file 12859_2018_2283_MOESM1_ESM.zip › phylotree.js-0.1.8/examples/vanilla/index.html]

```
d3.text("yokoyama.nwk", function(error, newick) {
  var height = 400,
    width = 400,

    tree = d3.layout.phylotree()
    .svg(d3.select("#tree_display"))
    .options({
      'left-right-spacing': 'fit-to-size',
      // fit to given size top-to-bottom
      'top-bottom-spacing': 'fit-to-size',
      // fit to given size left-to-right
      'selectable': false,
      // make nodes and branches not selectable
      'collapsible': false,
      // turn off the menu on internal nodes
      'transitions': false
      // turn off d3 animations
    })
    .size([height, width])
    .node_circle_size(0); // do not show "circles" at internal nodes

  tree(newick)
    // parse the Newick into a d3 hierarchy object with additional fields
    // generate node coordinates
    .layout();
  // render the tree

});
```
