# Supplementary material for: phylotree.js - a JavaScript library for application development and interactive data visualization in phylogenetics
Source: BMC Bioinformatics. 2018 Jul 25;19:276. doi: 10.1186/s12859-018-2283-2 (PMC6060545; doi:10.1186/s12859-018-2283-2)
Supplement: Supplementary file 1 — Latest release of source code. A zip file of the source code from release 0.1.8. Accessed 4 May 2018. (ZIP 3513 kb) [file 12859_2018_2283_MOESM1_ESM.zip › phylotree.js-0.1.8/examples/zoom/index.html]

Zoom using the mouse wheel, pan by clicking and dragging.

```
    var example_tree = "(((EELA:0.150276,CONGERA:0.213019):0.230956,(EELB:0.263487,CONGERB:0.202633):0.246917):0.094785,((CAVEFISH:0.451027,(GOLDFISH:0.340495,ZEBRAFISH:0.390163):0.220565):0.067778,((((((NSAM:0.008113,NARG:0.014065):0.052991,SPUN:0.061003,(SMIC:0.027806,SDIA:0.015298,SXAN:0.046873):0.046977):0.009822,(NAUR:0.081298,(SSPI:0.023876,STIE:0.013652):0.058179):0.091775):0.073346,(MVIO:0.012271,MBER:0.039798):0.178835):0.147992,((BFNKILLIFISH:0.317455,(ONIL:0.029217,XCAU:0.084388):0.201166):0.055908,THORNYHEAD:0.252481):0.061905):0.157214,LAMPFISH:0.717196,((SCABBARDA:0.189684,SCABBARDB:0.362015):0.282263,((VIPERFISH:0.318217,BLACKDRAGON:0.109912):0.123642,LOOSEJAW:0.397100):0.287152):0.140663):0.206729):0.222485,(COELACANTH:0.558103,((CLAWEDFROG:0.441842,SALAMANDER:0.299607):0.135307,((CHAMELEON:0.771665,((PIGEON:0.150909,CHICKEN:0.172733):0.082163,ZEBRAFINCH:0.099172):0.272338):0.014055,((BOVINE:0.167569,DOLPHIN:0.157450):0.104783,ELEPHANT:0.166557):0.367205):0.050892):0.114731):0.295021)"

    var tree = d3.layout.phylotree()
      .options({
        brush: false,
        zoom: true,
        "show-scale": false
      })
      .svg(d3.select("#tree_display"));

    tree(example_tree)
      .layout();
```
