# Supplementary material for: phylotree.js - a JavaScript library for application development and interactive data visualization in phylogenetics
Source: BMC Bioinformatics. 2018 Jul 25;19:276. doi: 10.1186/s12859-018-2283-2 (PMC6060545; doi:10.1186/s12859-018-2283-2)
Supplement: Supplementary file 1 — Latest release of source code. A zip file of the source code from release 0.1.8. Accessed 4 May 2018. (ZIP 3513 kb) [file 12859_2018_2283_MOESM1_ESM.zip › phylotree.js-0.1.8/index.html]

Toggle navigation

phylotree.js

- Newick 
  - Input Text

  - Export
- Examples 
  - HIV-1 RT
  - Unscaled IAV HA colored by host
  - NGS copy diversity
  - Compare NGS consensus
  - HIV-1 env multiple timepoints and compartments

Help
Documentation

Tag 

- New selection set
- Delete selection set
- Rename selection set


Cancel

Save

Selection 

- Add filtered nodes to selection
- Remove filtered nodes from selection
- Select all
- Select all internal nodes
- Select all leaf nodes
- Clear all internal nodes
- Clear all leaves
- Clear selection
- Label internal nodes using maximum parsimony
- Label internal nodes using conjunction (AND)
- Label internal nodes using disjunction (OR)

×

#### Newick string to render

(a : 0.1, (b : 0.11, (c : 0.12, d : 0.13) : 0.14) : 0.15)

Display this tree

Close

Linear

 Radial

Animation

Selected 0 and filtered 0 branches
